# No evidence for preferential X-chromosome inactivation as the main cause of divergent phenotypes in sisters with X-linked hypohidrotic ectodermal dysplasia

**DOI:** 10.1186/s13023-021-01735-2

**Published:** 2021-02-23

**Authors:** Laura Körber, Holm Schneider, Nicole Fleischer, Sigrun Maier-Wohlfart

**Affiliations:** 1 Center for Ectodermal Dysplasias and Department of Pediatrics, University Hospital Erlangen, Loschgestr. 15, 91054 Erlangen, Germany; 2 FDNA Inc, Boston, MA USA

**Keywords:** X-linked hypohidrotic ectodermal dysplasia, Ectodysplasin A, Female carriers, X-chromosome inactivation, Genotype–phenotype correlation

## Abstract

**Background:**

X-linked hypohidrotic ectodermal dysplasia (XLHED), a rare genetic disorder, affects the normal development of ectodermal derivatives, such as hair, skin, teeth, and sweat glands. It is caused by pathogenic variants of the gene *EDA* and defined by a triad of hypotrichosis, hypo- or anodontia, and hypo- or anhidrosis which may lead to life-threatening hyperthermia. Although female carriers are less severely affected than male patients, they display symptoms, too, with high phenotypic variability. This study aimed to elucidate whether phenotypic differences in female XLHED patients with identical *EDA* genotypes might be explained by deviating X-chromosome inactivation (XI) patterns.

**Methods:**

Six families, each consisting of two sisters with the same *EDA* variant and their parents (with either mother or father being carrier of the variant), participated in this study. XLHED-related data like sweating ability, dental status, facial dysmorphism, and skin issues were assessed. We determined the women`s individual XI patterns in peripheral blood leukocytes by the human androgen receptor assay and collated the results with phenotypic features.

**Results:**

The surprisingly large inter- and intrafamilial variability of symptoms in affected females was not explicable by the pathogenic variants. Our cohort showed no higher rate of nonrandom XI in peripheral blood leukocytes than the general female population. Furthermore, skewed XI patterns in favour of the mutated alleles were not associated with more severe phenotypes.

**Conclusions:**

We found no evidence for preferential XI in female XLHED patients and no distinct correlation between XLHED-related phenotypic features and XI patterns. Phenotypic variability seems to be evoked by other genetic or epigenetic factors.

## Introduction

X-chromosomal hypohidrotic ectodermal dysplasia (XLHED; MIM #305100) is a rare combined malformation of ectodermal tissues such as hair, teeth, and sweat glands, resulting in hypotrichosis, hypo-, oligo- or anodontia and hypo- or anhidrosis [1, 2]. The most crucial deficiency is the strongly reduced or missing ability to sweat, potentially leading to life-threatening hyperthermia, especially in infants [3]. Numerous pathogenic variants of the ectodysplasin A gene (*EDA*; NM_001399.4) are known to cause malfunctioning of the signaling protein EDA1 [4]. The presence of a certain single nucleotide polymorphism (SNP) rs3827760 (c.1109T>C; p.Val370Ala) in the gene *EDAR* (NM_022336.3; coding for the ectodysplasin A receptor) has been discussed to alleviate XLHED-related issues [5].

Beside the cardinal symptoms mentioned above, patients may show typical signs of facial dysmorphia (periorbital wrinkles and hyperpigmentation, frontal bossing, prominent lips, protruding ears). Due to the defective development of eccrine glands, individuals with XLHED often suffer from a very dry and/or eczematous skin, atrophic rhinitis, dry eyes, respiratory ailments, and in case of females, malformation of mammary glands [1,2,6–10].

Following an X-linked recessive inheritance pattern, normally only hemizygous males show the complete cluster of symptoms while heterozygous women were primarily considered as carriers. Although many females heterozygous for an *EDA* variant are indeed more mildly affected than male patients, they often do present distinct symptoms of pathological significance. They may lack numerous teeth and sweating ability [6] and, as a result of deficient breast development (which goes as far as uni- or bilateral amastia), difficulties with breastfeeding are experienced frequently [11].

Female XLHED patients with identical pathogenic *EDA* variants (e.g., sisters) occasionally present phenotypic features that vary in expression, which might be explained through the mechanism of preferential X-chromosome inactivation (XI). Usually, women show a random inactivation of one of the two X chromosomes in each somatic cell, which leads to an approximate 50:50 ratio of maternal to paternal X-chromosome expression (lyonization for dosage compensation) [12]. However, there are also women with a preferential or skewed XI, a phenomenon occasionally (but not exclusively) found in carriers of X-linked diseases. In these cases the ratio differs in favour of one of the two X-chromosomes, mostly that of the healthy wild-type allele, probably due to selective advantages of these cells. Cases of XLHED were discussed previously in this context [13–16].

So far, XLHED patients have been treated merely symptomatically. However, a new therapeutic approach appears to be very promising: the prenatal administration of a recombinant EDA1 molecule, which has already been applied to several boys (named patient use) resulting in normal sweating ability and improved dentition [17]. In order to evaluate this prenatal treatment, a clinical trial for yet unborn male patients is being prepared. The study presented here could lead to a better understanding of potential genotype–phenotype correlations in women with XLHED who have received only little scientific attention so far. These insights will most likely be of significance when it comes to the question whether affected females might also benefit from prenatal drug therapy.

## Subjects and methods

### Study design and patients

Six families (F1–F6), each consisting of two sisters (S1 and S2) and their parents (M, mother; one pair of siblings has different biological fathers), were included in this study. All sisters are carriers of pathogenic *EDA* variants with maternal inheritance in four and paternal inheritance in two of the familial cases.

### DNA analysis

DNA isolation from peripheral blood, polymerase chain reaction and subsequent Sanger sequencing were performed as described previously [18]. Specific oligonucleotide primer sequences and thermal cycling conditions for the detection of *EDA* and *EDAR* variants are available upon request.

### Clinical status and tooth quantification

Anthropometric data included body length in cm, weight in kg and body mass index (BMI) in kg/m^2^. HED-relevant issues (including general health, heat intolerance, dentition, skin and hair abnormalities) were assessed by questionnaires and physical examinations.

### Assessment of sweating ability

Quantification of pilocarpine-induced sweating (volumetry) in an area of 57 mm^2^ of the forearm for 30 min using the Wescor 3700 device (Wescor, Logan, USA) was performed as described before [17].

### Face2Gene analysis

Face2Gene (https://www.face2gene.com/), a next generation phenotyping tool developed by FDNA Inc. (Boston MA, USA), uses a facial recognition algorithm called DeepGestalt for evaluation of facial dysmorphisms. This technology quantifies similarities of facial frontal photographs of patients with hundreds of different syndromes the system has been trained on, resulting in a list of possible syndrome matches, ranked by a score called Gestalt Score (between 0 and 1) [19].

### HUMARA assay

The human androgen receptor (HUMARA) assay, a PCR-based method for the investigation of X-inactivation patterns, enables the discrimination between the maternal and paternal as well as the active and inactive X chromosome. A highly polymorphic trinucleotide repeat of exon 1 of the *HUMARA* gene allows to distinguish the two alleles (via their individual nucleotide length) and is informative in about 90% of all females. Cleavage sites for methylation sensitive restriction enzymes (HpaII and CfoI) close to the polymorphism allow the determination of the active (unmethylated) and the inactive (methylated) allele [20]. After restriction enzyme digestion of the DNA and subsequent PCR amplification with a fluorophore-labeled forward primer, fragment analysis was carried out using a Beckman CEQ-8800 sequencer. The calculations for determining XI patterns were performed as described previously [21]. XI ratios between 50:50 and 65:35% were scored as random, 66:34–80:20% as moderately skewed and > 80:20% as highly skewed [22]. Two samples with known XI patterns (a random one with a proportion of 50:50% and a nonrandom one with a proportion of 100:0%) were used as controls.

## Results

Although growth retardation and underweight are not rare among XLHED patients (male and female), anthropometric assessment of our cohort (mean age: 32.88, SD: 12.63) revealed no abnormalities (mean body length: 166.31 cm, SD: 4.76; mean body weight: 70.41 kg, SD: 14.75; mean BMI: 25.81, SD: 5.24) [23–25]. The mean number of deciduous teeth in the maxilla was 9.1 (SD: 1.37) and in the mandible 9.0 (SD: 1.63), however, data were not ascertainable in six out of 16 cases (38%). On average, the total number of permanent teeth in the upper jaw was 11.38 (SD: 2.28) and 10.19 (SD: 3.73) in the lower jaw. Except for one subject (F6_S1), all women were missing deciduous and/or permanent teeth, but with a high variability (Table 1).

**Table 1:** Anthropometric measurements and tooth quantification

Code	Age	Length (in cm)	Weight (in kg)	BMI	Number of deciduous teeth		Number of permanent teeth	
					Maxilla	Mandible	Maxilla	Mandible
F1_S1	16	167	52	19	8	6	12	10
F1_S2	19	165	70	26	9	10	13	14
F1_M	50	170	70	24	n/a	n/a	5	0
F2_S1	20	159	53	26	10	6	8	5 (+ 1 DT)
F2_S2	17	170	63	22	10	10	12	12
F2_M	44	163	68	26	10	10	11	11
F3_S1	40	165	115	42	n/a	n/a	11 (+ 1 DT)	9
F3_S2	37	157	70	28	n/a	n/a	14	11
F4_S1	23	170	60.5	21	6	9	10	11 (+ 2 DT)
F4_S2	21	165	55	20	8	9	11	13
F4_M	49	168	74	26	n/a	n/a	12	13
F5_S1	32	172	73	25	10	10	13	14
F5_S2	36	176	78	25	10	10	12	10
F6_S1	31	163	75	28	10	10	14	14
F6_S2	36	166	82	30	n/a	n/a	11	7
F6_M	55	165	68	25	n/a	n/a	13	9
Average	32.88	166.31	70.41	25.81	9.1	9.0	11.38	10.19
SD	12.63	4.76	14.75	5.24	1.37	1.63	2.28	3.73

*Abbreviations*: F, family; S, sister; M, mother; n/a, not available; DT, deciduous teeth; SD, standard deviation

56% of the affected women suffered from additional diseases, of which specific allergies, urticaria, neurodermatitis and eczema might be associated with their XLHED carrier status. Furthermore, six out  of 16 women (38%) reported to be photophobic and three (19%) had experienced recurrent conjunctivitis. Hearing impairments occurred in two (13%) of the patients (Table 2).

**Table 2:** Medical history

Code	Diseases other than XLHED	Recurrent eye problems		Hearing impairment
		Photophobia	Conjunctivitis	
F1_S1	Allergy	N	N	N
F1_S2	NAD	N	N	N
F1_M	NAD	N	N	N
F2_S1	Urticaria, neurodermatitis	Y	Y	Y
F2_S2	NAD	Y	N	N
F2_M	NAD	Y	N	N
F3_S1	Diabetes	Y	N	Y
F3_S2	NAD	Y	N	N
F4_S1	NAD	N	N	N
F4_S2	Hashimoto's thyroiditis	N	Y	N
F4_M	Hypothyroidism	N	Y	N
F5_S1	Glaucoma	Y	N	N
F5_S2	Hypothyroidism	N	N	N
F6_S1	Hypothyroidism	N	N	N
F6_S2	Allergy, eczema	N	N	N
F6_M	NAD	N	N	N

*Abbreviations*: F, family; S, sister; M, mother; NAD, no abnormality detected; Y, yes; N, no

XLHED-related phenotypic features were assessed by the Face2Gene facial recognition algorithm and by patient questionnaires during the annual family conference of the German-Swiss-Austrian ectodermal dysplasia patient organization or during visits at the Center for Ectodermal Dysplasias in Erlangen. Except for two patients (F1_S2 and F6_S1), XLHED was among the list of possible syndrome matches suggested by Face2Gene (*Gestalt Scores* ranging from 0.06 to 0.38; male XLHED patients usually show scores of approximately 1.0, data not published). XLHED was not one of the syndrome matches suggested by Face2Gene when analyzing the portrait pictures of unaffected mothers who served as negative controls.

Eleven out  of 16 subjects (69%) reported skin problems like dry and eczematous skin or neurodermatitis. Furthermore, 13 women showed sparse and/or thin scalp hair (81%), sparse eyebrows (81%) and in ten cases also sparse eyelashes (63%). Breast abnormalities, often associated with breastfeeding difficulties, were noted in twelve (75%) of the patients (Table 3).

**Table 3:** Phenotypic features

Code	Gestalt Score* (0─1)	Skin	Scalp hair	Eyebrows	Eyelashes	Breast
F1_S1	0.1	Partially dry	NAD	Sparse (esp. laterally)	NAD	Size asymmetry
F1_S2	u.a	NAD	NAD	NAD	NAD	NAD
F1_M	0.15	Dry	Sparse, thin	Very sparse	Very sparse	No mammary gland (left), no breastfeeding (voluntarily)
F2_S1	0.1	Neurodermatitis, dry scalp	Partially sparse	Sparse	Sparse	Soft mamillae
F2_S2	0.15	NAD	Thin, strawy	Sparse (esp. laterally)	Sparse (esp. laterally)	NAD
F2_M	0.12	Thin skin, partially dry	Sparse, bald spots (occipital)	Sparse	Sparse	Breastfeeding difficulties
F3_S1	0.16	Partially eczematous, dry	Sparse, bald spots (hairline and lateral)	Sparse (esp. laterally)	Sparse	Flat mamillae
F3_S2	0.13	Very dry	NAD	NAD	Sparse	Size asymmetry
F4_S1	0.16	NAD	Rather sparse	Sparse (esp. laterally)	NAD	NAD
F4_S2	0.37	Partially dry	Rather sparse	Sparse (esp. laterally)	NAD	NAD
F4_M	0.27	Dry	Sparse, thin	Rather sparse	NAD	Flat mamillae
F5_S1	0.06	Dry	Rather sparse	Rather sparse	Rather sparse	Breastfeeding difficulties
F5_S2	0.15	NAD	Rather sparse, thin	Sparse (esp. laterally)	Rather sparse	No breastfeeding, benign tumor
F6_S1	u.a	NAD	Partially sparse	NAD	NAD	Breastfeeding difficulties due to small mamillae
F6_S2	0.38	Eczematous (permanent steroid treatment)	Sparse	Sparse (esp. laterally)	Sparse	Breastfeeding difficulties
F6_M	0.15	Psoriasis	Partially sparse	Sparse	Sparse	Breastfeeding difficulties

*Abbreviations*: *, rounded; F, family; S, sister; M, mother; u.a., unable to assess; NAD, no abnormality detected; esp., especially

**Fig. 1:**
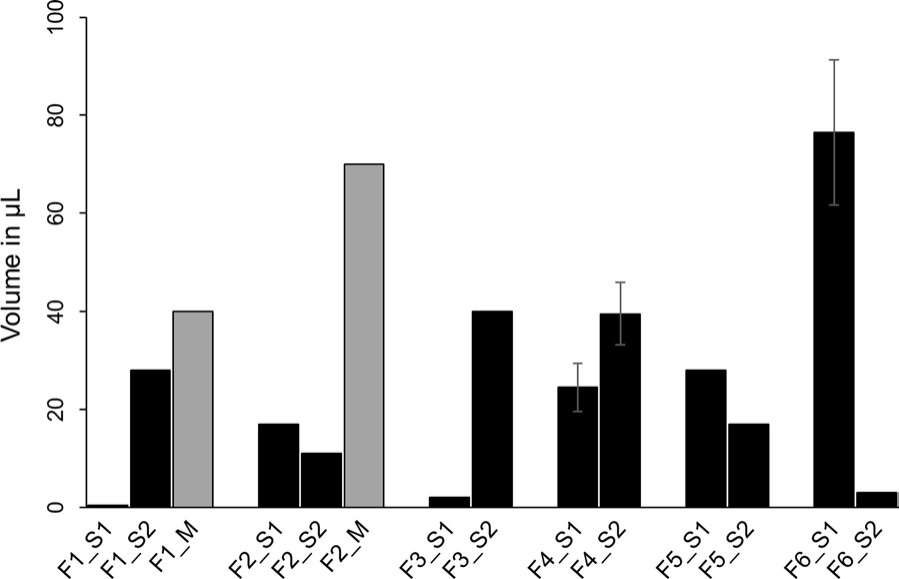
Quantification of pilocarpine-induced sweat production (volume in µL). Sisters are represented in black, mothers (whenever available for sweat collection) in grey

Heat intolerance was reported by six out  of 16 women (38%), although all subjects were able to sweat in at least some areas of their body. Hypohidrosis impaired daily life in four subjects (25%) and sporting activities in seven (44%) of the patients (Table 4). Pilocarpine-induced sweat volume on the forearm was 28.3 µl on average (SD: 23.5) and was ranging from complete anhidrosis (F1_S1) to normal sweating ability (Fig. 1; mean pilocarpine-induced sweat volume of healthy women: 61 μl [6]).

**Table 4:** Data related to sweating ability and heat intolerance

Code	Reported heat intolerance	Body areas with reported sweating ability	Reported impact of reduced sweating on:	
			Daily life	Sports
F1_S1	Y	Y	N	Y
F1_S2	N	Y	N	N
F1_M	N	Y	N	Y
F2_S1	Y	Y	Y	Y
F2_S2	N	Y	N	Y
F2_M	N	Y	N	N
F3_S1	Y	Y	Y	Y
F3_S2	N	Y	N	N
F4_S1	N	Y	N	N
F4_S2	N	Y	N	N
F4_M	Y	Y	N	N
F5_S1	N	Y	N	Y
F5_S2	N	Y	N	N
F6_S1	N	Y	N	N
F6_S2	Y	Y	Y	N
F6_M	Y	Y	Y	Y

*Abbreviations*: F, family; S, sister; M, mother; Y, yes; N, no

All familial variants identified in this study are known to be pathogenic and are associated with severe phenotypes in affected males (Table 5) [18,26–29]. None of the participants carried the potentially compensating SNP rs3827760 in the gene *EDAR*. The HUMARA assay revealed that eleven out  of 16 affected women (69%) display random, four (25%) moderately, and one (6%) highly skewed XI patterns. None of our female patients showed a complete inactivation of one of the two X chromosomes. Among the five cases with skewed XI, this was in favour of the wild-type allele (indicated by the higher XI ratio for the mutated allele) only in patient F4_M, while in the remaining four women (F3_S1, F5_S1, F6_S1 and F6_S2) the mutated allele was the preferentially active one (Table 5).

**Table 5:** *EDA* genotypes of affected individuals and X-chromosome inactivation patterns

Code	*EDA* variant	Changes at the amino acid level	Predicted effect	XI ratios in % (A1:A2)*	Classification
F1_S1	c.64_71dup8	p.Cys25AlafsX35	Truncated, dysfunctional protein, possibly causing NMD	**51**:49	Random
F1_S2	c.64_71dup8	p.Cys25AlafsX35	Truncated, dysfunctional protein, possibly causing NMD	**54**:46	Random
F1_M	c.64_71dup8	p.Cys25AlafsX35	Truncated, dysfunctional protein, possibly causing NMD	47:**53**	Random
F2_S1	c.917A>G	p.Gln306Arg	Impaired receptor binding	**37**:63	Random
F2_S2	c.917A>G	p.Gln306Arg	Impaired receptor binding	**51**:49	Random
F2_M	c.917A>G	p.Gln306Arg	Impaired receptor binding	**64**:36	Random
F3_S1	c.871G>A	p.Gly291Arg	Impaired receptor binding	**31**:69	Moderately skewed
F3_S2	c.871G>A	p.Gly291Arg	Impaired receptor binding	**65**:35	Random
F3_F	c.871G>A	p.Gly291Arg	Impaired receptor binding	/	/
F4_S1	c.467G>A	p.Arg156His	Abolished furin cleavage	**46**:54	Random
F4_S2	c.467G>A	p.Arg156His	Abolished furin cleavage	**39**:61	Random
F4_M	c.467G>A	p.Arg156His	Abolished furin cleavage	30:**70**	Moderately skewed
F5_S1	c.1045G>A	p.Ala349Thr	Impaired receptor binding	76:**24**	Moderately skewed
F5_S2	c.1045G>A	p.Ala349Thr	Impaired receptor binding	61:**39**	Random
F5_F	c.1045G>A	p.Ala349Thr	Impaired receptor binding	/	/
F6_S1	c.1133C>T	p.Thr378Met	Impaired receptor binding	81:**19**	Highly skewed
F6_S2	c.1133C>T	p.Thr378Met	Impaired receptor binding	71:**29**	Moderately skewed
F6_M	c.1133C>T	p.Thr378Met	Impaired receptor binding	**54**:46	Random

*Abbreviations*: F, family; S, sister; M, mother; NMD, nonsense-mediated decay; A1, allele 1 (the shorter allele); A2, allele 2 (the longer allele); *rounded mean values of the experiments using HpaII and CfoI, respectively; bold values mark the alleles with the disease-causing *EDA* variant

A comparison of the sweating abilities and XI patterns revealed no distinct correlation (limitations of this assessment are discussed later in the text). For example, the families F1 and F2 show distinct intrafamilial variations with regard to their sweating abilities, but these differences are not reflected by their XI status (all members with random XI ratios). Although patient F3_S1 with negligible sweat production was found to have skewed XI in favour of the mutation-carrying cells, the complete opposite was true for F6_S1, a woman with a similar XI pattern (also in favour of the mutated allele) but almost normal sweating ability.

## Discussion

One of the aims of this study was to gather and compare XLHED-related phenotypic features of females carrying pathogenic *EDA* variants, a group of patients who received only little scientific attention so far. Furthermore, we intended to find out whether variations in the expression of XLHED-related symptoms might be explained by deviating XI patterns as already reported for other X-linked diseases. In these cases, women escape the normally balanced mosaicism and show an increased expression level in one of the cell populations, or a decreased one, respectively.

The portion of patients with nonrandom XI patterns in our cohort is marginally higher than in the general female population, as approximately 10–30% (age-dependent) of unaffected women present skewed XI patterns (deviations of percentage rates found in the literature result from variable definitions of the threshold values for the XI ratios) [21,30–32]. The previously reported relation between the age of the women and higher skewing rates was not observed in our group of patients, which might be due to the limited cohort size.

We did not find a distinct correlation between disease manifestation and the XI pattern, concluding that XI is not the only explanation for phenotypic differences between female carriers of the same pathogenic *EDA* variant. Martínez-Romero et al. report similar results in their cohort of female XLHED patients in Spain [16]. Although XI patterns are usually comparable among the different tissues of the same individual, cases of tissue-specific discordance have been reported, too. Furthermore, this variability seems to be age-dependent and is particularly evident in women older than 60 years [32–35]. Peripheral blood leukocytes are commonly used for the investigation of selection mechanisms in XI-associated diseases, not only due to their easy accessibility but also to the high cell division rate. This leads to a higher selection pressure on cells carrying proliferation-inhibiting mutations and therefore on average to slightly higher rates of skewed XI [36, 37]. The XI patterns of blood and saliva in a Danish XLHED cohort showed a strong correlation [38]. Although determination of XI patterns in peripheral blood leukocytes by the HUMARA assay is a well-established procedure, there are limitations of the method and the current study. For example, the HUMARA assay is not informative in approximately 10% of the female population, so that other techniques have to be applied. In such cases, the XI status could be determined at the RNA level by analyzing the expression of informative X-linked polymorphisms [39]. Furthermore, tissue-specific variations of XI ratios cannot be excluded, but DNA from a sufficient number of skin biopsies or teeth is usually not available.

Cutaneous mosaicism, however, may present in different patterns, such as patches or lateralization, or following the linear lines of Blaschko. As we do not know whether the skin area where sweat was collected is affected or not, we cannot conclude much about the correlation between XI patterns and sweating ability.

Interestingly, several female patients reported about local differences (distinct from the lines of Blaschko) regarding the phenotypic expression, namely unilaterally reduced hair growth and sweating ability. The reason for lateralization, which has already been observed in other skin diseases, remains unclear [40].

Generally, skewed XI patterns can be evoked either by chance as a stochastic event, due to an impairment of the XI process itself (e. g. mutations in the XI-specific *XIST* gene) or because of selection mechanisms (the latter is hypothesized to occur more often in carriers of X-linked disorders [41, 42]. However, a selective advantage of one of the cell populations is always unidirectional and can therefore be excluded, as we found skewed XI ratios both in favour of the wild-type and the mutant allele.

The different pathogenic *EDA* variants of the six families are known to lead to comparable, full-blown clinical symptoms of XLHED in male patients. None of the patients carried the *EDAR* SNP rs3827760, a variant reported to potentially attenuate the severity of XLHED-related signs [5]. Nevertheless, other genetic or epigenetic factors, such as yet undetected polymorphisms, might have an impact on the patients’ phenotypes. They could be detected by future whole exome sequencing and subsequent genotype–phenotype association studies.

## Conclusions

Although we neither found higher rates of nonrandom XI in our patients nor a distinct correlation between skewed XI ratios and phenotypic expression levels, it is conceivable that in some cases extremely shifted levels might have protective or pathogenic effects, respectively. Our research accentuates the need for further large-cohort studies (comparing not only family members, but also unrelated female carriers of the same *EDA* genotype) for final conclusions. This, however, might be challenging because of the rarity of XLHED and the fact that asymptomatic carriers without affected male relatives usually remain undetected. Moreover, a determination of generally accepted criteria for the classification of XLHED phenotypes as mild, moderate or severe will be needed for more objective evaluations. This study is the first systematic approach to phenotyping in female XLHED patients, which might be relevant for the question whether a medical treatment option currently explored in clinical trials should also be considered for affected females.

## Data Availability

The datasets used and analysed during the current study are available from the corresponding author on reasonable request.

## Abbreviations

ED: Ectodermal dysplasia
EDA: Ectodysplasin A
HED: Hypohidrotic ectodermal dysplasia
HUMARA: The human androgen receptor assay
SNP: Single-nucleotide polymorphism
XI: X-chromosome inactivation
XLHED: X-linked hypohidrotic ectodermal dysplasia
